# Oxygen-enhanced MRI assessment of tumour hypoxia in head and neck cancer is feasible and well tolerated in the clinical setting

**DOI:** 10.1186/s41747-024-00429-1

**Published:** 2024-03-06

**Authors:** Alastair McCabe, Stewart Martin, Selene Rowe, Jagrit Shah, Paul S. Morgan, Damian Borys, Rafal Panek

**Affiliations:** 1 Academic Unit of Translational Medical Sciences, School of Medicine, University of Nottingham, Nottingham, UK; 2 Department of Clinical Oncology, Nottingham University Hospitals NHS Trust, City Hospital, Hucknall Road, Nottingham, NG5 1PB UK; 3 Department of Radiology, Nottingham University Hospitals NHS Trust, Nottingham, UK; 4 Mental Health & Clinical Neurosciences Unit, School of Medicine, University of Nottingham, Nottingham, UK; 5 Department of Medical Physics & Clinical Engineering, Nottingham University Hospitals NHS Trust, Nottingham, UK; 6 Department of Systems Biology and Engineering, Silesian University of Technology, Gliwice, Poland

**Keywords:** Head and neck neoplasms, Magnetic resonance imaging, Oxygen, Radiotherapy, Tumour hypoxia

## Abstract

**Background:**

Tumour hypoxia is a recognised cause of radiotherapy treatment resistance in head and neck squamous cell carcinoma (HNSCC). Current positron emission tomography-based hypoxia imaging techniques are not routinely available in many centres. We investigated if an alternative technique called oxygen-enhanced magnetic resonance imaging (OE-MRI) could be performed in HNSCC.

**Methods:**

A volumetric OE-MRI protocol for dynamic T1 relaxation time mapping was implemented on 1.5-T clinical scanners. Participants were scanned breathing room air and during high-flow oxygen administration. Oxygen-induced changes in T1 times (ΔT1) and *R*_2_* rates (Δ*R*_2_*) were measured in malignant tissue and healthy organs. Unequal variance *t*-test was used. Patients were surveyed on their experience of the OE-MRI protocol.

**Results:**

Fifteen patients with HNSCC (median age 59 years, range 38 to 76) and 10 non-HNSCC subjects (median age 46.5 years, range 32 to 62) were scanned; the OE-MRI acquisition took less than 10 min and was well tolerated. Fifteen histologically confirmed primary tumours and 41 malignant nodal masses were identified. Median (range) of ΔT1 times and hypoxic fraction estimates for primary tumours were -3.5% (-7.0 to -0.3%) and 30.7% (6.5 to 78.6%) respectively. Radiotherapy-responsive and radiotherapy-resistant primary tumours had mean estimated hypoxic fractions of 36.8% (95% confidence interval [CI] 17.4 to 56.2%) and 59.0% (95% CI 44.6 to 73.3%), respectively (*p* = 0.111).

**Conclusions:**

We present a well-tolerated implementation of dynamic, volumetric OE-MRI of the head and neck region allowing discernment of differing oxygen responses within biopsy-confirmed HNSCC.

**Trial registration:**

ClinicalTrials.gov, NCT04724096. Registered on 26 January 2021.

**Relevance statement:**

MRI of tumour hypoxia in head and neck cancer using routine clinical equipment is feasible and well tolerated and allows estimates of tumour hypoxic fractions in less than ten minutes.

**Key points:**

• Oxygen-enhanced MRI (OE-MRI) can estimate tumour hypoxic fractions in ten-minute scanning.

• OE-MRI may be incorporable into routine clinical tumour imaging.

• OE-MRI has the potential to predict outcomes after radiotherapy treatment.

**Graphical Abstract:**

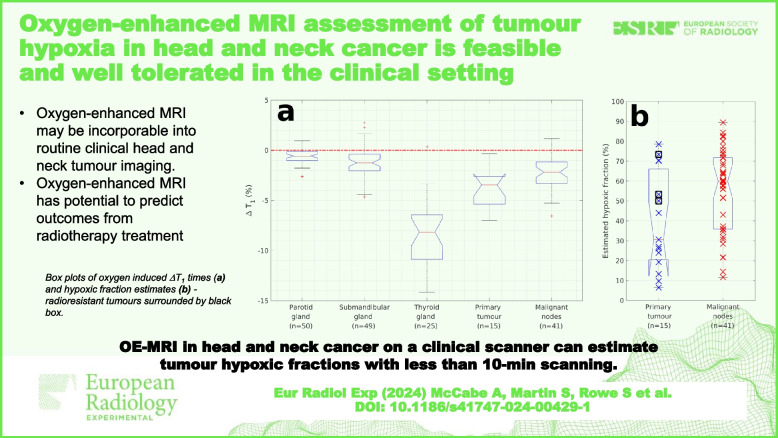

**Supplementary Information:**

The online version contains supplementary material available at 10.1186/s41747-024-00429-1.

## Background

Head and neck cancer is the eighth most common cancer in the UK [1] with the majority of tumours being head and neck squamous cell carcinomas (HNSCC). Survival rates vary by location with hypopharyngeal tumours having 5-year survival rates of only 27% [2]. Treatment approaches include surgical resection or radical radiotherapy with or without chemotherapy. Low levels of oxygen within malignant tissue (tumour hypoxia) are a well-recognised cause of local treatment failure in HNSCC treated with radical radiotherapy [3, 4]. Hypoxia modification strategies such as boost doses of radiation to hypoxic tumour regions [5, 6] have yet to garner routine clinical use in part due to the lack of readily accessible methods of determining and mapping tumour hypoxia [7, 8].

The most widely used hypoxia imaging technique is hypoxic positron emission tomography (PET), primarily performed with [^19^F]-labelled nitromidazole compounds [9]. Hypoxia-PET has been studied in HNSCC [10–16] and shown capable of predicting clinical outcomes [11, 13]; however, it requires an additional dedicated imaging session, can cost substantially more per scan than alternative clinical imaging techniques [17], and is not routinely available in most UK oncology centres.

Oxygen-enhanced magnetic resonance imaging (OE-MRI) is an alternative hypoxic imaging technique that maps tissue oxygenation based upon changes in the longitudinal relaxation rate R_1_ (1/T1) following the delivery of supplemental oxygen [18]. In well-oxygenated tissues, supplemental oxygen increases the concentration of dissolved oxygen due to the near-complete saturation of haemoglobin causing an increase in tissue *R*_1_ rates (decrease in T1 times) due to the paramagnetic properties of dissolved oxygen [19]. Conversely, in hypoxic tissues, additional oxygen preferentially alters the deoxyhaemoglobin to oxyhaemoglobin ratio with minimal alteration in plasma oxygen concentrations. T1 times in hypoxic regions are therefore relatively invariant to supplemental oxygen challenge [19–21].

Due to a lack of correlation between tumour averaged oxygen-induced T1 changes and reference hypoxia indicators [19, 21–25], previous OE-MRI studies have suggested combining OE-MRI with additional metrics such as tumour perfusion assessments [19] or blood oxygen level-dependent (BOLD) measurements [24, 26, 27]. BOLD refers to the reduction in *R*_2_* rate with the reduction in deoxyhaemoglobin to oxyhaemoglobin ratio as occurs in perfused hypoxic regions following an oxygen challenge. As *R*_1_ relaxation rates are also proportional to deoxyhaemoglobin concentrations [28], the addition of BOLD measurements to T1-based OE-MRI may refine the characterisation of the oxygenation status of tumours [24, 27].

The aims of this research were to assess if it is feasible to perform OE-MRI on a clinical scanner as part of a routine clinical imaging session in patients with HNSCC and if the addition of oxygen-induced Δ*R*_2_* measurements changes the categorisation of HNSCC oxygenation status. In addition, we sought to formally assess the tolerability of OE-MRI in patients with HNSCC and test differences between OE-MRI-derived parameters and clinical outcomes for patients treated with curative intent radiotherapy.

## Methods

All participants were prospectively recruited between August 2021 and November 2022 after research ethics approval (South Central Berkshire Research Ethics Committee, reference 21/SC/0050, 9 February 2021) and provided written informed consent (ClinicalTrials.gov identifier NCT04724096). Patient participants were identified prior to a clinically indicated neck MRI for the staging of suspected HNSCC by consultant surgeons on the national otolaryngology specialist register (minimum four-year experience). Histological diagnosis was not required prior to scanning if there was a strong clinical suspicion of HNSCC, reflecting the clinical diagnostic pathway at our institution. All primary tumours were confirmed as squamous cell carcinoma based on routine histopathological analysis of core biopsy specimens as per the clinical institutional procedures. Oropharyngeal primary tumours were tested for evidence of human papillomavirus (HPV) as per the local clinical protocols via *in situ* hybridisation for HPV deoxyribonucleic acid using the INFORM HPV III Family 16 Probe (Ventana Medical Systems, Tucson, AZ, USA).

Participants were scanned on Magnetom Sola 1.5-T scanners (Siemens Healthineers, Erlangen, Germany) wearing an adult nonrebreathing mask with a reservoir bag with the study protocol performed immediately prior to a routine clinical scan for patient participants. A safety valve allowed the wearing of the mask without supplemental gas delivery meaning patients could be switched from breathing room air to 15 L/min high-flow oxygen without requiring re-positioning or the use of a gas mixer. The posterior component of the head coil together with an ultraflex large 18-channel coil positioned over the neck region was used to facilitate the comfortable placement of the oxygen mask (Supplementary Fig. S1). Examinations were performed by state-registered clinically employed specialised radiographers (minimum one-year experience) as a component of patients’ routine clinical imaging appointments. Oxygen administration was supervised by a clinical oncologist (two-year experience).

**Fig. 1:**
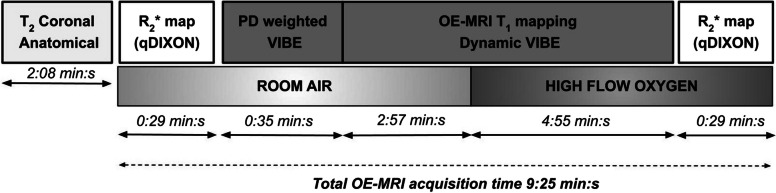
Study sequences and timing of switch from room air to high flow oxygen. Sequence parameters provided in Table 1. *OE-MRI* Oxygen-enhanced magnetic resonance imaging, *VIBE* Volumetric interpolated breath-hold examination

An initial T2-weighted sequence was performed to aid study sequence planning. All subsequent research sequences were acquired with identical field of view without repositioning the participant. *R*_2_* mapping was performed on room air and repeated after dynamic T1 mapping using a manufacturer-supplied multi-echo Dixon protocol (qDixon, Table 1). Dynamic T1 mapping was performed using the sequential acquisition of three-dimensional spoiled gradient echo volumetric interpolated breath-hold examination (VIBE) sequence. Following an image quality review after the first three nonpatient volunteers and two patients, the VIBE sequence was modified to increase the signal-to-noise ratio (Table 1). The resulting sequence was used for the subsequent 20 participants (2 nonpatient volunteers and 18 patients) with the overall OE-MRI acquisition taking 9:25 min:s (Fig. 1). The total air/oxygen breathing VIBE periods were 3:32 and 4:55 min:s, respectively. The VIBE temporal resolution was 11.8 s.

**Table 1:** Acquisition parameters for the study sequences

Sequence name	qDixon	VIBE
Sequence type	3D multi-echo Dixon	3D spoiled gradient-echo
Orientation	Axial	Axial
Repetition time (ms)	15.6	10 (4.2)
Echo time (ms)	Range 1.10 to 14.28 with 12 echoes	1.27 (1.48)
Flip angle (°)	4	2/18
Bandwidth (Hz/pixel)	1,090	399 (313)
Matrix	96 × 96	128 × 128
Field of view (mm^2^)	200 × 200	200 × 200
Slice thickness (mm)	2.5	2.5
Number slices	72	72
Number acquisitions	1 pre- and post-oxygen	Proton density: 3 (5) Dynamic: 40 (60)
Oxygen on (min:s)	-	3:32 (2:09)
Total acquisition time (min:s)	0:58	8:27 (5:35)
Time for single acquisition (s)	29	11.8 (5.15)

Values in parentheses represent the initial imaging protocol reviewed after the first three nonpatient volunteers and two patients

*3D* Three-dimensional

VIBE images were corrected for motion via nonrigid registration (using Advanced Normalization Tools [29]). The water image from the qDixon sequence was similarly registered for each participant and the resulting registration transformation was applied to derived qDixon parametric maps.

A four-dimensional median filter was applied to dynamic images before producing voxel-wise T1 maps for each dynamic acquisition using the variable flip angle−VFA methodology [30]. An initial room air period was defined from 0:35 to 3:32 min:s and hyperoxic phase from 5:30 min:s until the end of the dynamic sequence. Mean voxel-wise room air T1 times (T1Air) and hyperoxic T1 times (T1O_2_) were determined and voxel-wise changes in T1 times defined as:

$$\Delta T1\left(voxel\right)= \frac{T1{{{O}}}_{2}- T1{{Air}}}{T1{{Air}}} \times 100\%$$

Image processing was performed using in-house software developed in MATLAB version R2018b (Mathworks, Natick, MA, USA) by a clinical oncologist (two-year experience) and an MRI physicist (over ten-year experience). The stability of the T1 mapping sequence was assessed using a Eurospin TO5 phantom with twelve gel-filled tubes with varying T1 relaxation times [31]. T1 times were determined for volumes of interests (VOIs) on the central slice for each tube using identical methodology to the clinical experiments. Coefficients of variation over the study dynamic sequence were determined for each tube.

Histologically confirmed primary tumours were contoured on initial VIBE images. Malignant nodes were defined as nodal masses in patients with biopsy-proven HNSCC whose radiological appearance was deemed malignant by the local head and neck multidisciplinary team. Contoured volumes less than 0.25 mL were excluded. Parotid, submandibular, and thyroid glands were contoured in accordance with radiotherapy contouring guidelines [32]. All contouring was performed using ITK-SNAP (version 3.4.0) [33] by a clinical oncologist (two-year experience).

Average VOI ΔT1 times and Δ*R*_2_* rates were defined as the median of $\Delta T1\left(voxel\right)$ times and voxel *R*_2_* rate differences with statistical significance against the null hypothesis of no change in median value assessed via Wilcoxon signed-rank test, with *p*-values lower than 0.05 as significant. Individual voxels within VOIs were interrogated for significant ΔT1 via a two-tailed unequal variance *t*-test, with *p*-values lower than 0.05 as significant. Estimates of hypoxic fractions were defined as the percentage of voxels not showing statistically significant negative ΔT1. Parcellation analysis of primary tumours and malignant nodes was performed using VOI averaged ΔT1 times and Δ*R*_2_* rates [24]. Statistical analysis was performed using MATLAB version R2018b by a clinical oncologist (two-year experience) and an MRI physicist (over ten-year experience).

All patient participants were surveyed on their scan experience using an MRI-specific anxiety questionnaire [34]. Patients were asked to complete two copies of the questionnaire pertaining to the study protocol and routine clinical scan. Paired scores were compared using the Wilcoxon signed-rank test, with *p*-values lower than 0.05 as significant.

Patients with histologically confirmed HNSCC were discussed at the local head and neck multidisciplinary meeting and received treatment at a single centre in accordance with local protocols. Twelve weeks following the completion of curative intent radiotherapy, patients underwent an ^18^F-fluoro-2-deoxy-D-glucose-PET scan [35]. Primary tumours with a complete metabolic response were classed as responders. Nonresponders had subsequent management decided upon by the local head and neck multidisciplinary team. Patients’ treatment and follow-up were unaffected by the study. Estimated hypoxic fractions and VOI averaged ΔT1 were compared for responding and nonresponding groups via two-tailed unequal variance *t*-test, with *p*-values lower than 0.05 as significant.

## Results

Temporal stability assessment of the dynamic VIBE T1 mapping protocol showed median (range) coefficients of variation 0.2% (0.1 to 0.4%) (Supplementary Fig. S2).

Five nonpatient volunteers (median age 32 years, range 31 to 60) and 20 patients with suspected HNSCC (median age 57 years, range 36 to 76) were recruited of whom 15 received histological diagnoses of HNSCC (median age 59 years, range 38 to 76). Out of the 5 patients who did not have HNSCC, 1 had a lymphoepithelial cyst (patient 2), 1 had benign cystic lesion (patient 18), and 3 had no radiological abnormality identified (patients 8, 12, 17). Adequate inflation of the mask nonrebreather bag was recorded for all participants. One patient (patient ID 12) terminated the study early following dynamic acquisition 32. ΔT1 maps were produced with the hyperoxic phase defined using the final seven dynamic measurements.

### Normal structures

**Fig. 2:**
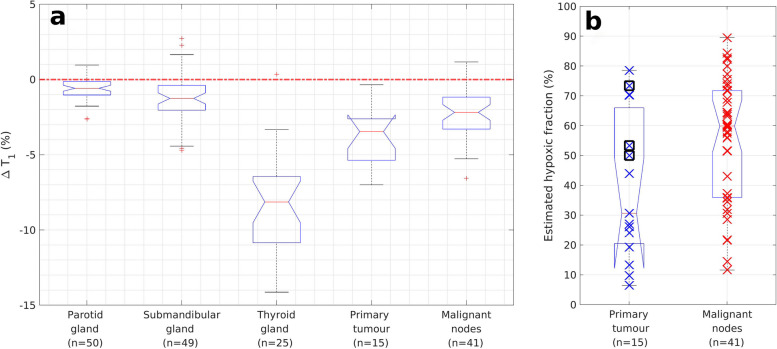
**a** Notched box plots of oxygen-induced ΔT1 (%) times for all 25 participants for the parotid, submandibular, and thyroid glands and for the 15 patients with head and neck squamous cell carcinoma for the primary tumour and malignant nodal masses. The single outlier point showing positive ΔT1 in the thyroid gland came from a nonpatient volunteer. **b** Notched box plots with overlaid data points of estimates of hypoxic fractions (%) for malignant tissues. Data points corresponding to primary tumours with evidence of residual disease post-radiotherapy treatment are surrounded by a black box

**Fig. 3:**
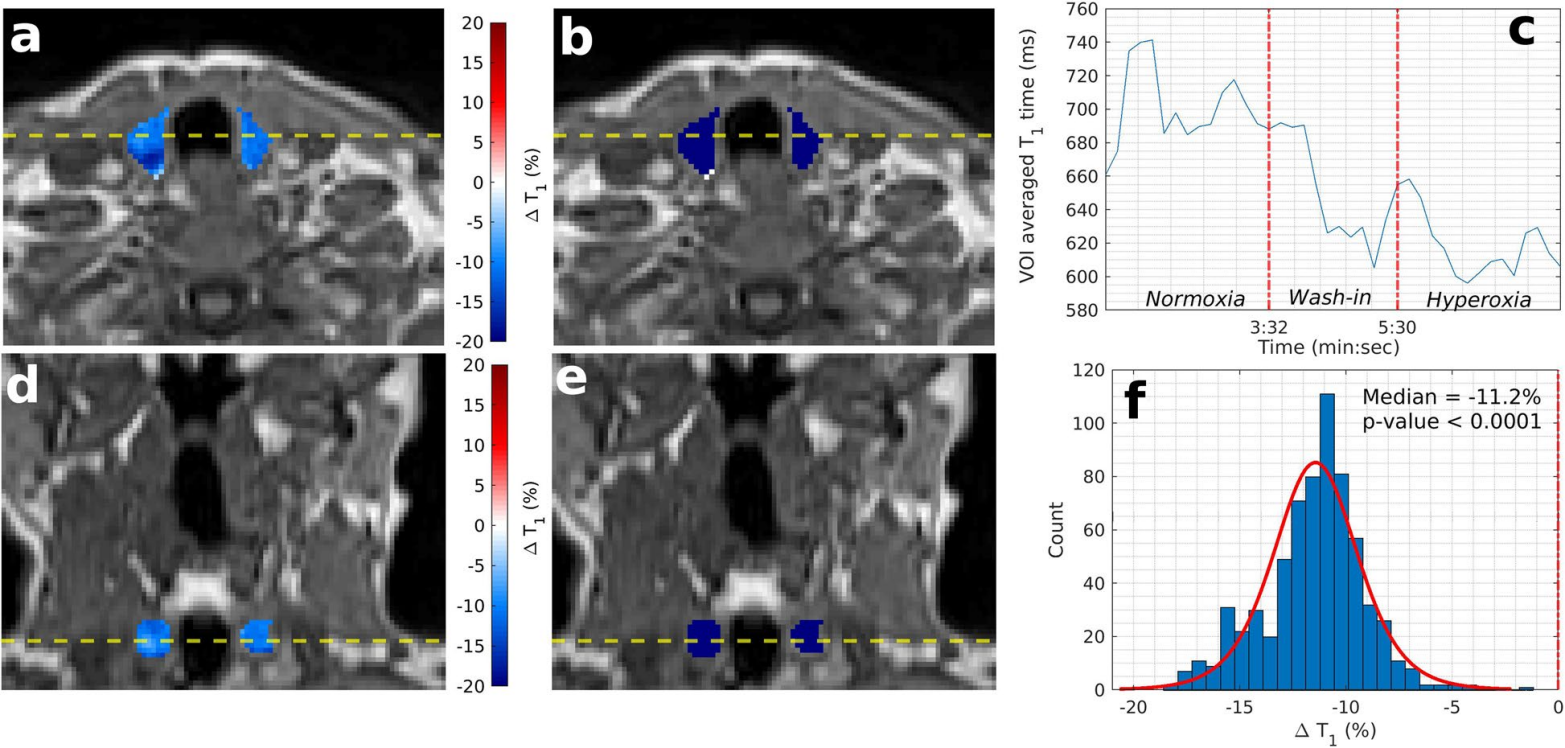
Example OE-MRI parametrical maps of thyroid gland (patient number 5). T1 weighted VIBE image is shown with overlaid parametric ΔT1 map of the thyroid gland (**a**, **d**, axial and coronal plane respectively) and overlaid statistical map of ΔT1 times (**b**, **e**). Blue colour indicates statistically significant decrease in T1 times, white indicates no statistically significant change, and red indicates statistically significant increasing T1 times. **c** Time series of T1 times averaged over the entire thyroid VOI. **f** Histogram of ΔT1 times for the entire thyroid VOI. *OE-MRI* Oxygen-enhanced magnetic resonance imaging, *VIBE* Volumetric interpolated breath-hold examination, *VOI* Volume of interest

OE-MRI data from all 25 participants was used to determine VOI averaged ΔT1 times for normal structures in the head and neck region. Median (range) ΔT1 times were -0.6% (-2.6 to 1.0%) for parotid glands, -1.3% (-4.7 to 2.7%) for submandibular glands and -8.1% (-14.1 to 0.4%) for thyroid glands (Fig. 2). Figure 3 shows example ΔT1 parametric maps, VOI averaged T1 time series, and total VOI ΔT1 histogram for an example thyroid gland.

### Malignant tissue

Fifteen primary tumours (median volume 5.04 mL, range 0.54 to 33.70 mL) and 41 distinct malignant nodal masses (median volume 2.62 mL, range 0.26 to 20.56 mL) were contoured (Table 2).

**Table 2:** Characteristics of the squamous cell carcinomas for the 15 patients with histologically confirmed tumours

Patient number	**Sex**	**Age (years)**	**Smoking**	**Site**	**Stage (TNM8)**	**p16**	**HPV DNA**	**Primary size (m**L**)**	**Lymph node masses**	
									Number	Size (mL) Median (range)
**1**	M	54	Never	Palatine tonsil	T4aN2b	-	N/A	31.15	2	1.90 (1.31–2.50)
**3**	M	45	Current	Pyriform fossa	T2N2b	N/A	N/A	4.35	2	7.04 (4.27–9.81)
**4**	M	69	Ex	Oropharynx	T0N2	+	+	-	1	10.71
**5**	M	66	Never	Pyriform fossa	T2N1	+	+	3.98	2	6.26 (1.64–10.88)
**6**	M	46	Current	Nasopharynx	T1N2	N/A	N/A	8.83	10	2.60 (1.01–20.56)
**7**	M	66	Ex	Base of tongue	T4aN2	+	N/A	33.70	4	1.83 (0.37–6.44)
**9**	M	62	Never	Base of tongue	T1N1	+	+	1.31	1	11.98
**10**	M	55	Current	Base of tongue	T3N1	+	+	5.04	2	2.90 (0.26–5.55)
				Palatine tonsil	T3N0	+	+	17.69	−	−
**11**	M	72	Ex	Pyriform fossa	T4aN1	N/A	N/A	33.11	1	0.46
**13**	F	59	Current	Vallecula	T1N2c	-	-	0.54	1	9.94
**14**	M	56	Current	Palatine tonsil	T4aN2b	+	-	21.60	3	1.10 (0.48–8.40)
**15**	M	38	Current	Palatine tonsil	T1N1	+	+	2.35	7	2.62 (0.54–7.19)
**16**	M	65	Never	Palatine tonsil	T2N1	+	N/A	3.75	2	6.00 (0.93–11.07)
**19**	F	76	Never	Palatine tonsil	T2N1	+	+	4.55	1	8.35
**20**	M	57	Current	Larynx	T3N2b	N/A	N/A	7.20	2	1.52 (1.17–1.87)
		Median (range)						5.04 (0.26–33.70)	2 (1–10)	2.62 (0.26–20.56)
		Total number of lesions						15	41	-

All histology and staging were discussed at the local head and neck cancer multidisciplinary team meeting

*HPV DNA* Human papillomavirus deoxyribonucleic acid, *N/A* Not assessed

**Fig. 4:**
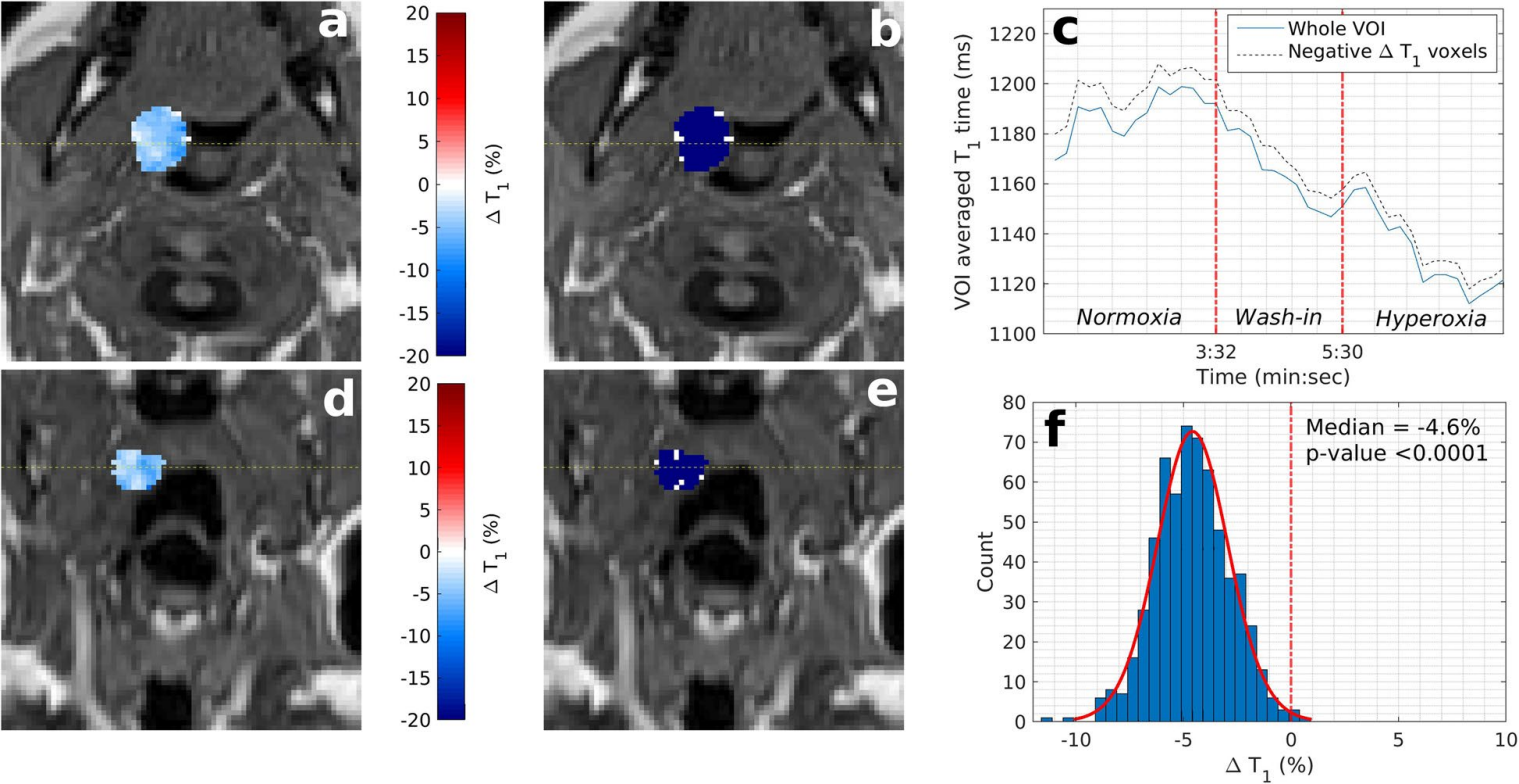
Example OE-MRI parametrical maps in a primary tumour with a complete response to radiotherapy treatment (patient number 16). Low estimated hypoxic fraction (9.8%) is shown on a T1-weighted VIBE image with overlaid parametric ΔT1 map of the primary tumour (**a**, **d**, axial and coronal plane, respectively) and overlaid statistical map of ΔT1 times (**b**, **e**). Blue colour indicates a statistically significant decrease in T1 times, white indicates no statistically significant change, and red indicates statistically significant increasing T1 times. **c** Time series of T1 times averaged over the entire primary tumour VOI and over those voxels with significantly decreasing T1 times only. **f** Histogram of ΔT1 times for the entire primary tumour VOI. *OE-MRI* Oxygen-enhanced magnetic resonance imaging, *VIBE* Volumetric interpolated breath-hold examination, *VOI* Volume of interest

**Fig. 5:**
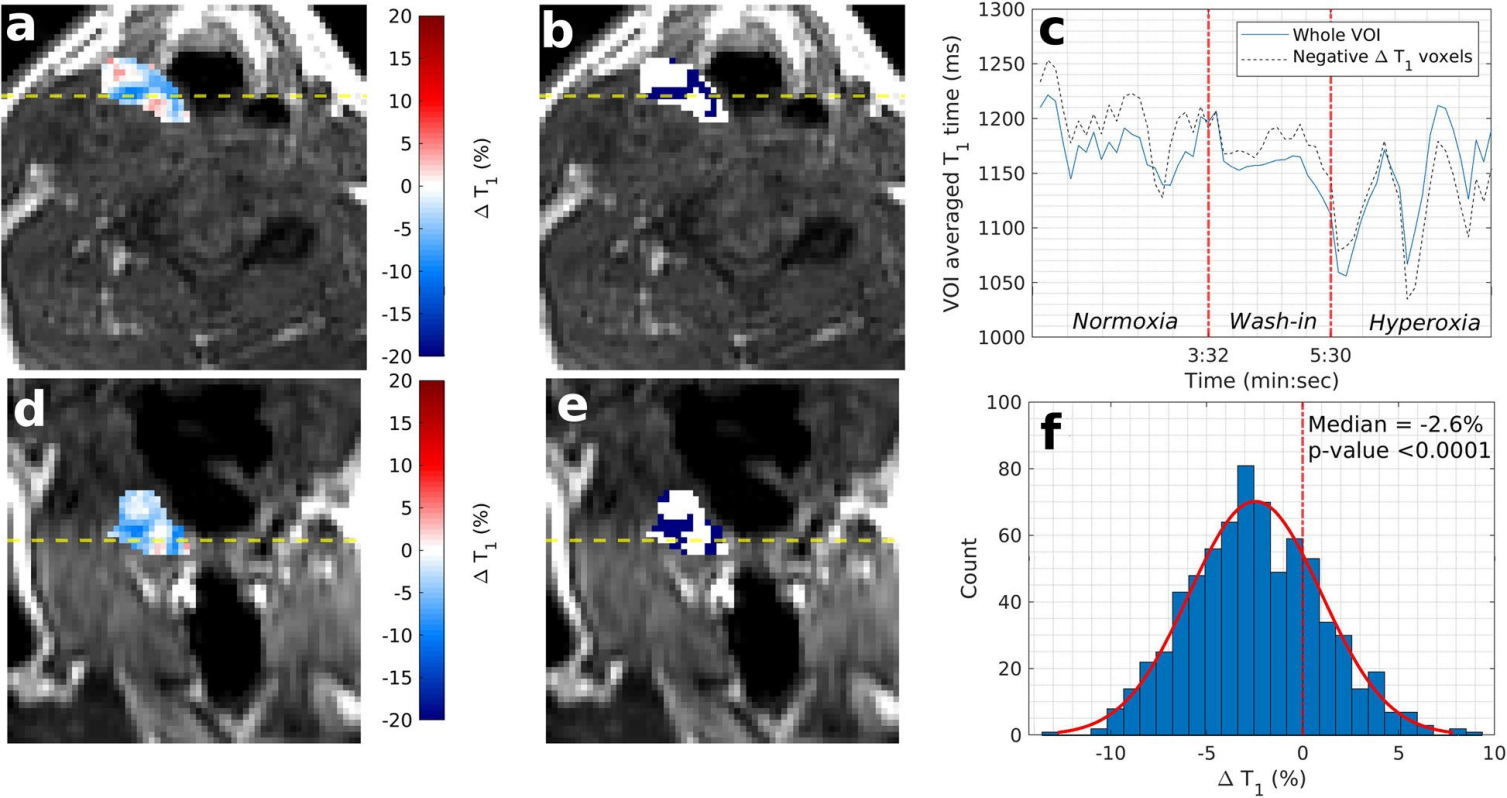
Example OE-MRI parametrical maps in a patient with a primary tumour with residual disease post radiotherapy treatment (patient number 3). High estimated hypoxic fraction (73.5%) is shown on a T1-weighted VIBE image with overlaid parametric ΔT1 map of the primary tumour (**a**, **d**, axial and coronal plane, respectively) and overlaid statistical map of ΔT1 times (**b**, **e**). Blue colour indicates a statistically significant decrease in T1 times, white indicates no statistically significant change, and red indicates statistically significant increasing T1 times. **c** Time series of T1 times averaged over the entire primary tumour VOI and over those voxels with significantly decreasing T1 times only. **f** Histogram of ΔT1 times for the entire primary tumour VOI. *OE-MRI* Oxygen-enhanced magnetic resonance imaging, *VIBE* Volumetric interpolated breath-hold examination, *VOI* Volume of interest

Median (range) ΔT1 times were -3.5% (-7.0 to -0.3%) for primary tumours and -2.2% (-6.5 to 1.2%) for malignant nodes. Median (range) estimated hypoxic fractions for primary tumours and malignant nodes were 30.7% (6.5 to 78.6%) and 59.9% (11.6 to 89.5%) respectively (Fig. 2). HPV-related and unrelated oropharyngeal cancers had median (range) estimated hypoxic fractions of 25.6% (6.5 to 70.2%) and 52.4% (26.1 to 78.6%) respectively. Figures 4 and 5 show example ΔT1 parametric maps, VOI averaged T1 time series, and total VOI ΔT1 histograms for example primary tumours with low and high estimated hypoxic fractions respectively (full data in Additional file 1: Appendix 1).

Out of 15 patients with confirmed HNSCC, 11 (73%) proceeded to curative intent radiotherapy and 9 underwent post-treatment ^18^F-fluoro-2-deoxy-D-glucose-PET scan (1 patient had post-treatment MRI scan). Of these 10 primary tumours, 7 (70%) were responders, with 3 (30%) showing residual disease, confirmed histologically in 2 cases (Table 3). Mean estimated hypoxic fractions were 36.8% (95% confidence intervals [CI] 17.4 to 56.2%) and 59.0% (95% CI 44.6 to 73.3%) for responding and nonresponding primary tumours respectively (*p* = 0.111, unequal variance *t*-test). VOI mean ΔT1 values were -3.8% (95% CI -5.6 to -2.0%) for responding and -3.1% (95% CI -3.5 to -2.6%) for residual disease (*p* = 0.484, unequal variance *t*-test).

**Table 3:**
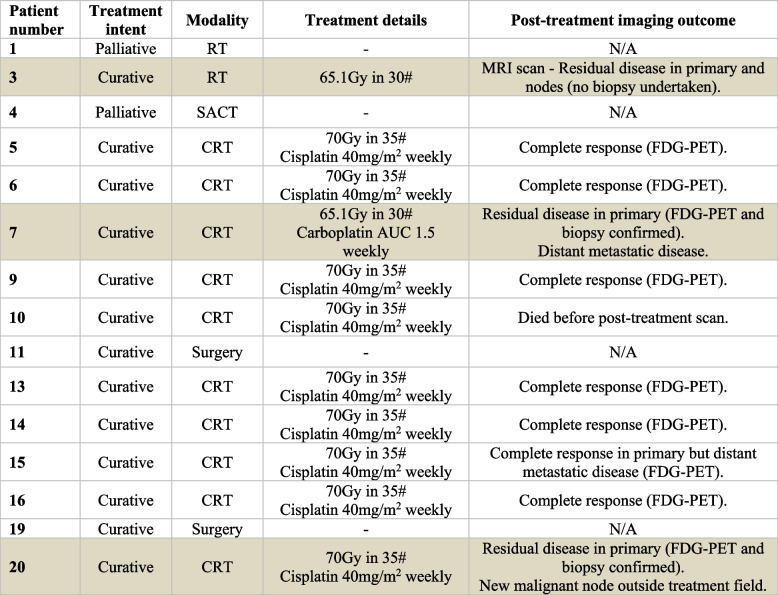
Treatment details and outcomes of post-treatment imaging for the 15 patient participants with histologically confirmed squamous cell carcinoma

Radiotherapy dose presented is delivered to the region containing the primary tumour. Post-treatment imaging was completed for 10 patients with one of them having an MRI scan rather than an FDG-PET scan due to the clinical circumstances. Highlighted rows indicate those patients with residual disease in the primary tumour on post-treatment imaging

*CRT* Concurrent chemoradiotherapy, *FDG-PET*^18^F-fluorodeoxyglucose positron emission tomography, *RT* Radiotherapy, *SACT* Systemic anticancer therapy

**Fig. 6:**
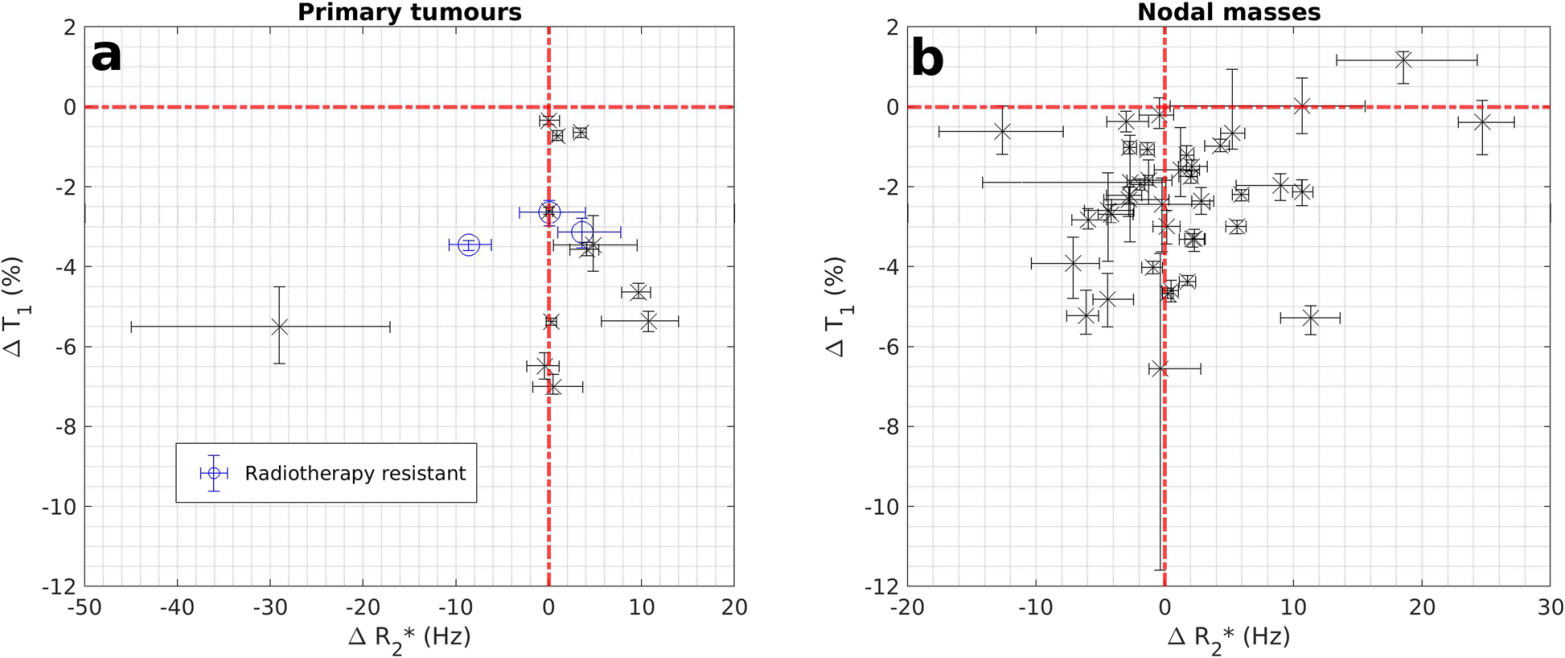
Scatter plot showing categorisation of hypoxia status of primary tumours (**a**) and malignant nodes (**b**) based on VOI average median ΔT1 times and Δ*R*_2_* rates with error bars indicating estimates of 95% confidence intervals derived from Bootstrap sampling with 1,000 samples. Data points corresponding to primary tumours with evidence of residual disease post-radiotherapy treatment are shown with blue circles. *VOI* Volume of interest

*R*_2_* measurements were obtained in all participants. Median (range) Δ*R*_2_* rates were 0.4 Hz (-29.1 to 10.7 Hz) for primary tumours and 0.2 Hz (-12.7 to 24.7 Hz) for malignant nodes (Fig. 6). In patients who had curative intent radiotherapy, mean Δ*R*_2_* rates were 6.3 Hz (95% CI 1.9 to 10.7 Hz) and -1.7 Hz (95% CI -8.8 to 5.4 Hz) for responding and radiotherapy-resistant primary tumours (*p* = 0.812, unequal variance *t*-test) with baseline mean *R*_2_* rates of 33.3 Hz (95% CI 15.0 to 51.5 Hz) and 44.8 Hz (95% CI 42.3 to 47.3 Hz) respectively (*p* = 0.897, unequal variance *t*-test).

### Anxiety questionnaire

**Fig. 7:**
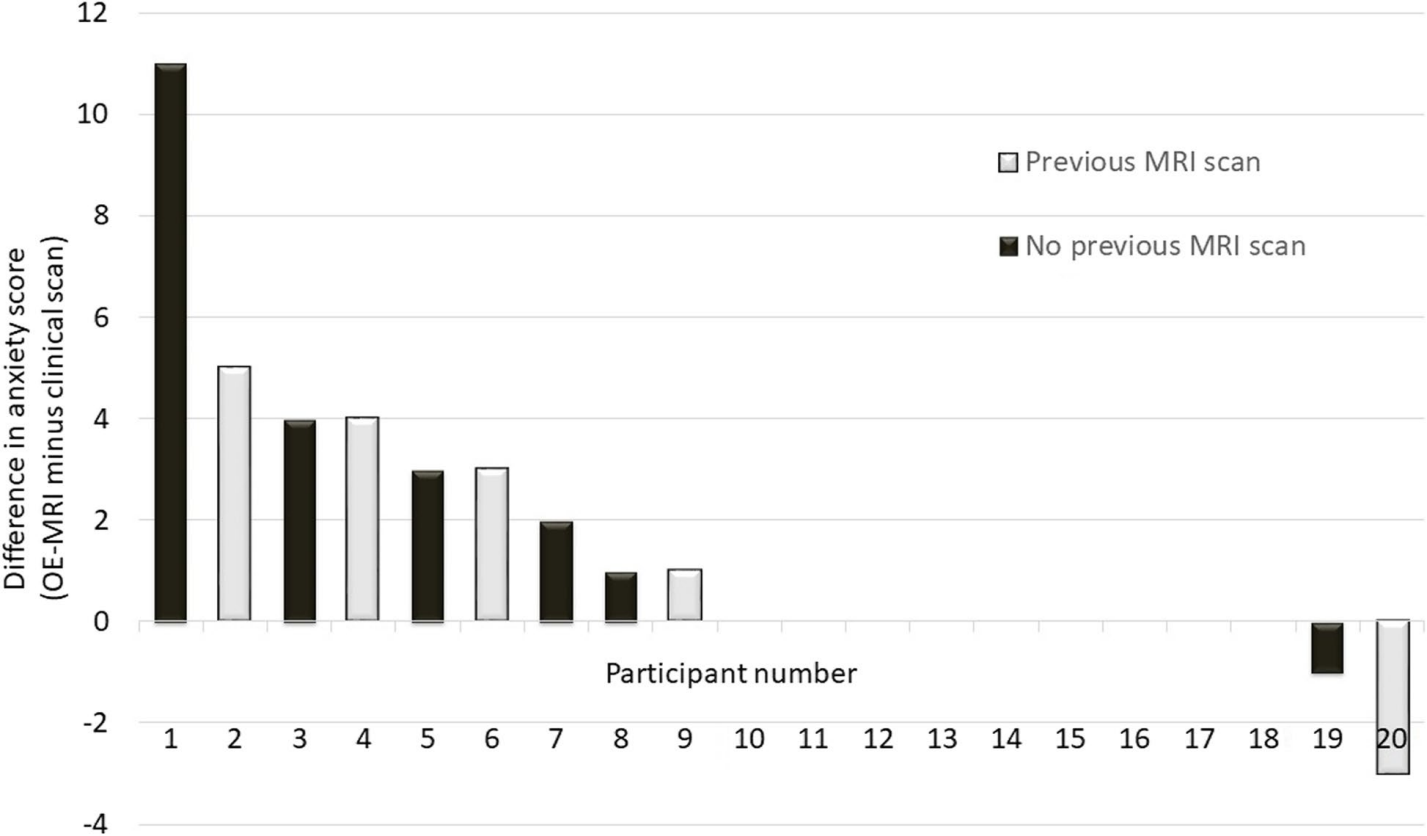
Waterfall plot showing the difference in individual participant anxiety scores between the OE-MRI scan and the routine clinical scan grouped by whether they had undergone a previous scan (of any anatomical region). Higher scores indicate worse anxiety. *OE-MRI* Oxygen-enhanced magnetic resonance imaging

All 20 patients completed the anxiety questionnaire with 40% (8/20) stating they had undergone an MRI scan previously. Questionnaire scores can range between 15 (lowest anxiety) and 60 (maximal anxiety) [36]. Median anxiety scores were 19.5 (range 15 to 42) and 15.5 (range 15 to 45) for study and routine scans respectively (*p* = 0.026 Wilcoxon signed rank). The mean difference in anxiety scores (study minus clinical scan) was +1.5 (standard deviation 3.0, Fig. 7 and Supplementary Table S1).

## Discussion

Our OE-MRI protocol was designed to be added to a standard clinical protocol using routinely available equipment therefore making this a low-cost and rapidly translatable method of hypoxia imaging in HNSCC. For this reason, we elected to use room air as opposed to medical air for the pre-oxygen acquisitions in order to avoid the need for a medical gas supply and additional equipment in the form of gas mixers. Although this meant participants did not have the opportunity to become accustomed to the gas delivery before oxygen was commenced, we did not encounter any apparent disadvantages from this approach. Despite the OE-MRI sequence taking less than ten minutes, it was possible to image the entire tumour volume and discern differing responses to supplemental oxygen challenge.

### Normal structures

The magnitude of oxygen-induced T1 shortening in parotid and submandibular glands is small indicating limited sensitivity of the OE-MRI technique in these structures. In contrast, the thyroid gland showed strong and consistent negative ΔT1. As OE-MRI relies on the absence of T1 shortening to determine hypoxic regions, a clinically relevant quality control structure to ensure adequate oxygen delivery to organs is desirable [18, 21, 25]. We suggest the thyroid gland may represent such a structure in the head and neck.

### Malignant tissue

There is limited data in the literature on oxygen-induced ΔT1 times in patients with HNSCC. A single study at 1.5 T reported mean oxygen-induced Δ*R*_1_ rates of 0.019/s in six patients with HNSCC on a diagnostic MRI system, corresponding to ΔT1 of -2.1% which is comparable to the median primary tumour VOI ΔT1 of -3.5% in this study. All 15 primary tumours in the current study showed statistically significant shortening of VOI median ΔT1 times mirroring the observed oxygen-induced prolongation of all VOI averaged ΔR_1_ rates in the previous 1.5-T study [37]. In contrast, a study at 3-T in five patients with oropharyngeal cancers reported tumour Δ*R*_1_ rates ranging from -0.001 to 0.108/s with two of the mean baseline *R*_1_ rates failing to show statistically significant change with oxygen [38].

Although our study and the previous two studies all used volumetric acquisitions, the 3-T study by Bluemke et al. [38] made single T1 measurements before and after oxygen to determine tumour Δ*R*_1_ rates whereas our study and that of the previous 1.5-T study by Dubec et al. [37] used dynamic acquisitions with multiple T1 measurement time points before and after oxygen administration. These dynamic measurements allow voxel-wise oxygen-induced ΔT1/Δ*R*_1_ maps to be produced following image coregistration to control for participant motion. Bluemke et al. [38] acknowledge that patient motion was a significant difficulty encountered in their study and focus their OE-MRI analysis on describing oxygen-induced changes to tumour region of interest *R*_1_ histograms. We feel that dynamic OE-MRI acquisitions with appropriate image registration allow greater characterisation of tumour oxygenation status; however, standardisation of imaging parameters and data processing approaches is clearly required for ready comparison of OE-MRI studies [39] as has been developed in other quantitative MRI techniques [40, 41].

The presence of hypoxia in HNSCC is well documented with oxygenation levels measured invasively using Eppendorf oxygen tension (pO_2_) histography showing overall median tumour pO_2_ of 10 mmHg corresponding to median hypoxic fractions of 21% and 32% depending upon the threshold set to define radiobiologically significant hypoxia (2.5 mmHg and 5 mmHg, respectively) [42]. PET-based imaging assessments of tumour hypoxia in HNSCC have shown significant interlesion variability with individual tumour fractional hypoxia estimates ranging from 0 to 95% and study population median values ranging from 0.9 to 66% [10–12, 16]. In our study, estimated hypoxic fractions also show significant inter-lesion variability but the median estimated hypoxic fraction in primary tumours of 31% is comparable to direct measurements.

In our study, radiotherapy nonresponding tumours show a 29.3% greater average hypoxic fraction than responding tumours, albeit not statistically significant potentially due to the sample size of this pilot study. There was no difference found with VOI averaged ΔT1 times between responding and nonresponding tumours. This is in keeping with OE-MRI studies in other tumour sites which have failed to identify correlations between spatially averaged changes in T1/R_1_ values and reference hypoxia markers [39]. Hypoxia-imaging biomarkers that capture information pertaining to the distribution of hypoxic regions are therefore likely to prove the most clinically relevant.

The dichotomisation of a voxel’s hypoxic status based on the lack of statistically significant T1 shortening is clearly an oversimplification of the underlying continuously varying oxygen tension present in tumours. The relationship between T1 times and tumour pO_2_ is dependent on a number of physical and physiological variables meaning it is not possible to readily ascribe pO_2_ values to imaged voxels [28]. Consequently, it is not possible to state the effective pO_2_ threshold for the estimates of hypoxic fractions presented in this study. The magnitude of our hypoxic fractions may be overestimated due to the inclusion of any cystic, necrotic, or nonperfused areas in the VOI [19] as well as a consequence of the study power returned from performing individual voxel analysis on a limited number of dynamic data points. This power could be increased at the expense of scan time or with the use of higher scan acceleration.

The OE-MRI sequence was developed for in-plane and slice resolution to be adequate to facilitate the fusion of acquired parametric maps to radiotherapy planning computed tomography scans with the ambition of mapping tumour hypoxia distributions to assist in radiotherapy volume delineation. Although the spatial resolution used in this study (1.6 × 1.6 × 2.5 mm^3^) is higher than other clinical OE-MRI studies [39], it is still significantly coarser than the typical diffusion distance of molecular oxygen (~100 μm [8]). Although the image resolution used here may detect greater levels of variability in oxygen distribution compared to coarser acquisitions, this comes at the cost of a worse signal-to-noise ratio, which may reduce the ability to discern borderline normoxic voxels thus overestimating tumour hypoxic burden.

It has been suggested that negative oxygen-induced Δ*R*_2_* rates may occur in hypoxic tumours due to decreases in deoxyhaemoglobin concentrations with supplemental oxygen and could potentially discriminate lower levels of hypoxia from normoxia in tumours with oxygen-induced T1 shortening [24, 27]. We identified two primary tumours that showed such a decrease in Δ*R*_2_* rates but only one of the three radiotherapy-resistant HNSCC primary tumours had such a change. In addition, we did not identify any statistically significant difference in *R*_2_* parameters between radiotherapy-responsive and radiotherapy-resistant primary tumours. The relationship between oxygen-induced ΔT1 times, Δ*R*_2_* rates, and baseline *R*_2_* is known to be complex, in part due to potential spatial fluctuations in perfusion. As such, spatially averaged Δ*R*_2_* rates may not be sensitive enough to offer useful insights on tumour hypoxia.

### Anxiety questionnaire

Undertaking OE-MRI in patients with HNSCC may cause greater distress than imaging in other anatomical regions due to the nature of the symptoms commonly experienced by this patient group. In addition, as hypoxic-PET imaging studies have suggested that hypoxic regions that fail to reoxygenate with treatment are a more sensitive biomarker of radioresistance [11, 13], repeated OE-MRI assessments during treatment may be beneficial [13, 43]. Although it has been demonstrated previously that OE-MRI is tolerated by patients with HNSCC [37], a formal assessment of patients’ experience of the OE-MRI scan to ensure maximal tolerability and therefore willingness to undergo repeat scanning is useful.

Overall, the OE-MRI scan was well tolerated with only one participant terminating their study scan prematurely. Although anxiety scores were worse with the OE-MRI scan, the magnitude of the difference was small and of doubtful clinical significance; however, all participants voluntarily agreed to undergo a longer MRI scan and therefore potentially represent a subset of patients less prone to MRI scan anxiety. In addition, as the study sequence was performed prior to routine clinical imaging, participants may have felt more comfortable with the MRI environment as the imaging session progressed. Further OE-MRI studies should be mindful of the potential burden additional or extended scans place on this group of patients who are undergoing an already significant amount of medical imaging during a psychologically challenging time.

### Limitations

The aim of this study was to evaluate the feasibility of performing OE-MRI in suspected HNSCC in a clinical environment and not to repeat the work of previous authors in demonstrating the efficacy of OE-MRI in determining tumour hypoxia [19, 21, 25, 36, 44]. Nevertheless, histopathological correlation with a suitable hypoxia marker would have enabled comparison and evaluation of the accuracy of the estimated hypoxic fractions. In addition, at our centre, routine histological verification of malignant cervical lymph nodes is reserved for cases with equivocal radiological findings only. Without surgical treatment, no definitive histology of nodal masses is obtained hence correlation with clinical outcome in this study was limited to histologically confirmed primary tumours.

The majority of primary tumours in this study were oropharyngeal (11/15) with 82% (9/11) positive for p16 expression (high-risk HPV variant deoxyribonucleic acid detected in 7 cases) representing a likely selection bias in the patient recruitment design of the study. HPV-related cancers are relatively more radiosensitive than HPV-negative tumours, and although both types display similar levels of hypoxia [45], pharmacological hypoxia modification therapies appear less effective in HPV-positive tumours [46, 47]. The ability to stratify HPV-positive tumours based on their hypoxia status may therefore be less clinically relevant than for HPV-negative tumours.

## Conclusions

We present a well-tolerated implementation of dynamic, volumetric OE-MRI in the head and neck region using routinely available equipment in an acute hospital setting that was able to discern differing responses to supplemental oxygen within biopsy-confirmed HNSCC. Further adequately powered studies are now required in HNSCC to investigate the predictive power of OE-MRI estimated tumour hypoxic fractions in discriminating patients more likely to benefit from the addition of hypoxia modification therapy to radiotherapy treatments.

### Supplementary Information

**Additional file 1: Appendix 1.** Tumour characteristics and OE-MRI derived parameters for all identified primary tumours and malignant nodal masses. **Fig. S1.** A member of the research team modelling the participant setup. The posterior component of the head coil was used together with an ultraflex large 18-channel coil positioned over the neck region allowing ready placement of the non-rebreather oxygen mask. The non-rebreather bag was positioned on top of the ultraflex coil. Study participants wore ear plugs and ear defenders and did not wear a surgical face mask during the scan. **Fig. S2.** T1 values for 12 tubes in a Eurospin TO5 phantom measured using the study dynamic vibe sequence. T1 values quoted are mean values over all 40 acquisitions. Coefficient of variations quoted for each tube have a median value of 0.2%. **Fig. S3.** Example OE-MRI parametrical maps in a patient with a suspected malignant nodal mass (patient no: 16 Lymph Node no: 2). Low estimated hypoxic fraction (11.6%) is shown on a T1 weighted vibe image with overlaid parametric ΔT1 map of the malignant mass (a, d, axial and coronal plane respectively) and overlaid statistical map of ΔT1 times (b, e). Blue colour indicates statistically significant decrease in T1 times, white indicating no statistically significant change and red indicating statistically significant increasing T1 times. c Time series of T1 times averaged over the entire malignant node VOI and over those voxels with significantly decreasing T1 times only. f Histogram of ΔT1 times for the entire malignant nodal VOI.* OE-MRI* Oxygen-enhanced magnetic resonance imaging, *VIBE *Volumetric interpolated breath-hold examination, *VOI* Volume of interest. **Fig. S4.** Example OE-MRI parametrical maps in a patient with a suspected malignant nodal mass (patient no: 15 Lymph Node no: 7). High estimated hypoxic fraction (72.0%) is shown on a T1 weighted vibe image with overlaid parametric ΔT1 map of the malignant mass (a, d, axial and coronal plane respectively) and overlaid statistical map of ΔT1 times (b, e). Blue colour indicates statistically significant decrease in T1 times, white indicating no statistically significant change and red indicating statistically significant increasing T1 times. c Time series of T1 times averaged over the entire malignant node VOI and over those voxels with significantly decreasing T1 times only. f Histogram of ΔT1 times for the entire malignant nodal VOI.* OE-MRI* Oxygen-enhanced magnetic resonance imaging, *VIBE *Volumetric interpolated breath-hold examination, *VOI* Volume of interest. **Supplementary Table S1.** Summed scores from all patient participants for each of the 15 domains assessed in the MRI specific anxiety questionnaire.

## Data Availability

All data generated or analysed during this study are included in this published article and its supplementary information files.

## Abbreviations

BOLD: Blood oxygen level-dependent
CI: Confidence interval
HNSCC: Head and neck squamous cell carcinoma
HPV: Human papillomavirus
OE-MRI: Oxygen-enhanced magnetic resonance imaging
PET: Positron emission tomography
pO_2_: Oxygen tension
VIBE: Volumetric interpolated breath-hold examination
VOI: Volume of interest
