# Supplementary material for: Oxygen-enhanced MRI assessment of tumour hypoxia in head and neck cancer is feasible and well tolerated in the clinical setting
Source: Eur Radiol Exp. 2024 Mar 6;8:27. doi: 10.1186/s41747-024-00429-1 (PMC10914657; doi:10.1186/s41747-024-00429-1)
Supplement: Supplementary file 1 — Additional file 1: Appendix 1. Tumour characteristics and OE-MRI derived parameters for all identified primary tumours and malignant nodal masses. Fig. S1. A member of the research team modelling the participant setup. The posterior component of the head coil was used together with an ultraflex large 18-channel coil positioned over the neck region allowing ready placement of the non-rebreather oxygen mask. The non-rebreather bag was positioned on top of the ultraflex coil. Study participants wore ear plugs and ear defenders and did not wear a surgical face mask during the scan. Fig. S2. T1 values for 12 tubes in a Eurospin TO5 phantom measured using the study dynamic vibe sequence. T1 values quoted are mean values over all 40 acquisitions. Coefficient of variations quoted for each tube have a median value of 0.2%. Fig. S3. Example OE-MRI parametrical maps in a patient with a suspected malignant nodal mass (patient no: 16 Lymph Node no: 2). Low estimated hypoxic fraction (11.6%) is shown on a T1 weighted vibe image with overlaid parametric ΔT1 map of the malignant mass (a, d, axial and coronal plane respectively) and overlaid statistical map of ΔT1 times (b, e). Blue colour indicates statistically significant decrease in T1 times, white indicating no statistically significant change and red indicating statistically significant increasing T1 times. c Time series of T1 times averaged over the entire malignant node VOI and over those voxels with significantly decreasing T1 times only. f Histogram of ΔT1 times for the entire malignant nodal VOI. OE-MRI Oxygen-enhanced magnetic resonance imaging, VIBE Volumetric interpolated breath-hold examination, VOI Volume of interest. Fig. S4. Example OE-MRI parametrical maps in a patient with a suspected malignant nodal mass (patient no: 15 Lymph Node no: 7). High estimated hypoxic fraction (72.0%) is shown on a T1 weighted vibe image with overlaid parametric ΔT1 map of the malignant mass (a, d, axial and coronal plane respectively) [file 41747_2024_429_MOESM1_ESM.docx]

**Oxygen-enhanced MRI assessment of tumour hypoxia in head and neck cancer is feasible and well tolerated in the clinical setting**

**ELECTRONIC SUPPLEMENTARY MATERIAL**

**Appendix 1**

| PRIMARY TUMOUR | | | | T1 time: median (ms) | | | | R_2_* rate: median (Hz) | | |
| --- | --- | --- | --- | --- | --- | --- | --- | --- | --- | --- |
| Participant | **p16** | **HPV DNA** | **Volume (cm^3^)** | **Baseline**  **(IQR)** | **ΔT1 (%)**  **(IQR)** | **p-value** | **Hypoxic fraction (%)** | **Baseline**  **(IQR)** | **ΔR_2_***  **(IQR)** | **p-value** |
| 1 | - | N/A | 31.15 | 1417  (294) | -0.7  (4.8) | <0.0001 | 78.6 | 26.1  (27.6) | 0.9  (16.9) | 0.0004 |
| 3 | N/A | N/A | 4.35 | 1170  (197) | -2.6  (4.7) | <0.0001 | 73.5 | 43.6  (35.2) | 0.0  (37.0) | 0.6852 |
| 5 | + | + | 3.98 | 1239  (198) | -5.4  (4.2) | <0.0001 | 30.7 | 27.8  (39.1) | 10.7  (38.8) | <0.0001 |
| 6 | N/A | N/A | 8.83 | 1400  (178) | -0.6  (2.5) | <0.0001 | 70.4 | 20.6  (26.9) | 3.4  (14.7) | <0.0001 |
| 7 | + | N/A | 33.70 | 1206  (239) | -3.4  (4.9) | <0.0001 | 50.0 | 47.3  (66.6) | -8.6  (55.5) | <0.0001 |
| 9 | + | + | 0.61 | 716  (152) | -3.5  (2.7) | <0.0001 | 44.0 | 51.4  (39.2) | 4.8  (18.1) | 0.0392 |
| 10 (i) | + | + | 5.04 | 1034  (190) | -7.0  (3.8) | <0.0001 | 13.3 | 68.8  (57.7) | 0.4  (27.6) | 0.5291 |
| 10 (ii) | + | + | 17.69 | 997  (196) | -2.6  (2.3) | <0.0001 | 27.1 | 26.1  (23.3) | 0.0  (8.1) | 0.0115 |
| 11 | N/A | N/A | 33.11 | 1211  (223) | -5.4  (4.4) | <0.0001 | 19.3 | 25.1  (25.4) | 0.2  (25.4) | 0.2705 |
| 13 | - | - | 0.54 | 817  (372) | -5.5  (6.0) | <0.0001 | 26.1 | 144.8  (73.1) | -29.1  (49.7) | <0.0001 |
| 14 | + | - | 21.60 | 1066  (146) | -0.3  (4.3) | <0.0001 | 70.2 | 29.6  (17.7) | 0.0  (17.7) | 0.5403 |
| 15 | + | + | 2.35 | 1206  (173) | -6.5  (3.6) | <0.0001 | 6.5 | 21.7  (27.0) | -0.5  (15.2) | 0.4338 |
| 16 | + | N/A | 3.75 | 1200  (194) | -4.6  (2.3) | <0.0001 | 9.8 | 33.6  (20.5) | 9.7  (17.4) | <0.0001 |
| 19 | + | + | 4.55 | 1256  (164) | -3.6  (3.0) | <0.0001 | 24.1 | 20.1  (22.2) | 4.1  (18.0) | <0.0001 |
| 20 | N/A | N/A | 7.20 | 1179  (224) | -3.1  (6.4) | <0.0001 | 53.4 | 43.5  (40.5) | 3.6  (45.8) | <0.0001 |

| MALIGNANT NODES | | | | T1 time: median (ms) | | | | R_2_* rate: median (Hz) | | |
| --- | --- | --- | --- | --- | --- | --- | --- | --- | --- | --- |
| Participant | **p16** | **HPV DNA** | **Volume (cm^3^)** | **Baseline**  **(IQR)** | **ΔT1 (%)**  **(IQR)** | **p-value** | **Hypoxic fraction (%)** | **Baseline**  **(IQR)** | **ΔR_2_***  **(IQR)** | **p-value** |
| 1 (i) | - | N/A | 2.50 | 1353  (296) | -1.8  (5.6) | <0.0001 | 64.6 | 29.6  (12.7) | -1.3  (13.5) | 0.1550 |
| 1 (ii) | - | N/A | 1.31 | 1087  (216) | 1.2  (4.1) | 0.0008 | 82.8 | 11.2  (32.2) | 18.5  (31.9) | <0.0001 |
| 3 (i) | N/A | N/A | 4.27 | 1262  (429) | -3.0  (6.0) | <0.0001 | 63.3 | 21.3  (10.1) | 0.1  (10.9) | 0.6329 |
| 3(ii) | N/A | N/A | 9.81 | 1327  (302) | -3.0  (4.5) | <0.0001 | 60.2 | 17.5  (15.1) | 5.6  (14.9) | <0.0001 |
| 4 (i) | + | + | 10.71 | 1339  (629) | -1.0  (4.6) | <0.0001 | 60.5 | 15.2  (18.1) | 4.3  (17.3) | <0.0001 |
| 5 (i) | + | + | 10.88 | 1343  (194) | -2.2  (3.1) | <0.0001 | 59.9 | 14.3  (8.8) | 5.9  (9.8) | <0.0001 |
| 5(ii) | + | + | 1.64 | 1177  (197) | -2.3  (3.2) | <0.0001 | 51.7 | 30.9  (19.9) | -2.8  (19.7) | 0.0684 |
| 6 (i) | N/A | N/A | 7.23 | 1547  (244) | -1.7  (2.4) | <0.0001 | 60.3 | 20.1  (14.3) | 2.0  (10.5) | <0.0001 |
| 6 (ii) | N/A | N/A | 3.50 | 1426  (146) | -1.5  (2.9) | <0.0001 | 56.1 | 20.0  (13.2) | 2.1  (11.3) | <0.0001 |
| 6 (iii) | N/A | N/A | 20.56 | 1674  (619) | -1.8  (3.7) | <0.0001 | 67.9 | 15.2  (15.8) | -1.3  (7.8) | <0.0001 |
| (iv) | N/A | N/A | 1.01 | 847  (654) | -2.6  (7.8) | <0.0001 | 63.9 | 28.1  (28.7) | -4.4  (8.7) | <0.0001 |
| 6 (v) | N/A | N/A | 1.16 | 1324  (433) | -0.2  (3.1) | 0.5021 | 77.4 | 20.8  (12.4) | -0.4  (6.3) | 0.4582 |
| (vi) | N/A | N/A | 8.41 | 1345  (198) | -4.0  (3.2) | <0.0001 | 37.3 | 17.0  (7.7) | -1.0  (12.5) | <0.0001 |
| 6 (vii) | N/A | N/A | 1.19 | 1402  (203) | -2.2  (2.1) | <0.0001 | 43.1 | 16.5  (8.4) | -2.8  (7.6) | <0.0001 |
| 6 (viii) | N/A | N/A | 1.70 | 1455  (101) | -2.7  (2.7) | <0.0001 | 31.9 | 15.2  (11.2) | -4.2  (7.7) | <0.0001 |
| 6 (ix) | N/A | N/A | 6.32 | 1424  (142) | -1.9  (2.3) | <0.0001 | 51.5 | 18.2  (7.8) | -2.0  (8.4) | <0.0001 |
| 6 (x) | N/A | N/A | 1.54 | 1327  (363) | -1.2  (3.4) | <0.0001 | 73.4 | 24.5  (14.1) | 1.7  (7.0) | <0.0001 |
| 7 (i) | + | N/A | 0.37 | 1333  (392) | -1.6  (4.2) | 0.0045 | 80.0 | 20.6  (12.3) | 1.2  (7.5) | 0.0827 |
| 7 (ii) | + | N/A | 3.13 | 1371  (220) | -5.3  (3.8) | <0.0001 | 34.6 | 16.4  (15.0) | 11.3  (19.5) | <0.0001 |
| 7 (iii) | + | N/A | 6.44 | 1232  (288) | -0.4  (5.1) | <0.0001 | 84.4 | 29.8  (14.5) | -3.0  (21.3) | <0.0001 |
| 7 (iv) | + | N/A | 0.54 | 903  (383) | -1.9  (6.8) | 0.0059 | 74.2 | 17.5  (22.4) | -2.7  (22.1) | 0.0080 |
| 9 (i) | + | + | 11.98 | 1258  (269) | -4.7  (3.7) | <0.0001 | 14.5 | 18.9  (12.8) | 0.2  (7.6) | 0.0314 |
| 10 (i) | + | + | 5.55 | 1019  (254) | -4.6  (3.6) | <0.0001 | 21.7 | 18.1  (10.6) | 0.5  (7.1) | 0.0104 |
| 10 (ii) | + | + | 0.26 | 577  (187) | -6.5  (12.5) | 0.0001 | 28.6 | 23.8  (12.5) | -0.4  (4.6) | 0.4074 |
| 11 (i) | N/A | N/A | 0.46 | 957  (231) | -3.9  (5.9) | <0.0001 | 36.0 | 27.7  (17.5) | -7.1  (7.6) | <0.0001 |
| 13 (i) | - | - | 9.94 | 1567  (252) | -1.1  (3.4) | <0.0001 | 69.0 | 12.7  (12.2) | -1.4  (10.8) | <0.0001 |
| 14 (i) | + | - | 8.40 | 1246  (177) | -3.3  (3.7) | <0.0001 | 35.7 | 16.4  (13.1) | 2.1  (13.7) | <0.0001 |
| 14 (ii) | + | - | 0.48 | 1205  (365) | -2.4  (4.8) | <0.0001 | 59.5 | 20.2  (11.6) | -0.2  (12.4) | 0.6045 |
| 14 (iii) | + | - | 1.10 | 1202  (211) | -0.6  (4.4) | 0.1290 | 89.5 | 23.0  (17.5) | -12.7  (29.4) | <0.0001 |
| 15 (i) | + | + | 5.93 | 1454  (303) | -2.8  (4.1) | <0.0001 | 58.0 | 16.6  (11.7) | -6.0  (13.8) | <0.0001 |
| 15 (ii) | + | + | 0.54 | 1546  (330) | -4.8  (3.5) | <0.0001 | 21.6 | 9.1  (7.5) | -4.5  (6.7) | 0.0002 |
| 15 (iii) | + | + | 2.62 | 1590  (289) | -2.1  (3.5) | <0.0001 | 55.9 | 6.9  (7.3) | 10.6  (8.0) | <0.0001 |
| 15 (iv) | + | + | 0.62 | 1521  (267) | -2.0  (2.7) | <0.0001 | 64.4 | 15.2  (12.3) | 9.0  (7.4) | <0.0001 |
| 15 (v) | + | + | 3.40 | 1656  (384) | -3.3  (3.4) | <0.0001 | 30.7 | 18.4  (11.0) | 2.3  (7.4) | <0.0001 |
| 15 (vi) | + | + | 0.93 | 1664  (320) | -0.6  (3.1) | 0.0012 | 71.7 | 20.2  (6.4) | 5.2  (4.0) | <0.0001 |
| 15 (vii) | + | + | 7.19 | 1577  (318) | -1.0  (2.7) | <0.0001 | 72.0 | 18.4  (11.0) | -2.8  (6.4) | <0.0001 |
| 16 (i) | + | N/A | 0.93 | 1187  (351) | -5.2  (5.0) | <0.0001 | 21.7 | 27.1  (12.0) | -6.1  (5.8) | <0.0001 |
| 16 (ii) | + | N/A | 11.07 | 1249  (148) | -4.4  (2.6) | <0.0001 | 11.6 | 24.1  (9.7) | 1.7  (10.6) | <0.0001 |
| 19 (i) | + | + | 8.35 | 1448  (585) | -2.3  (6.6) | <0.0001 | 57.5 | 14.4  (18.7) | 2.8  (11.7) | <0.0001 |
| 20 (i) | N/A | N/A | 1.87 | 1359  (257) | -0.4  (6.3) | 0.8391 | 75.6 | 17.3  (19.9) | 24.7  (16.3) | <0.0001 |
| 20 (ii) | N/A | N/A | 1.17 | 1285  (240) | 0.0  (6.2) | 0.6378 | 82.3 | 10.8  (25.2) | 10.7  (45.2) | 0.0004 |

**Supplementary Figures**


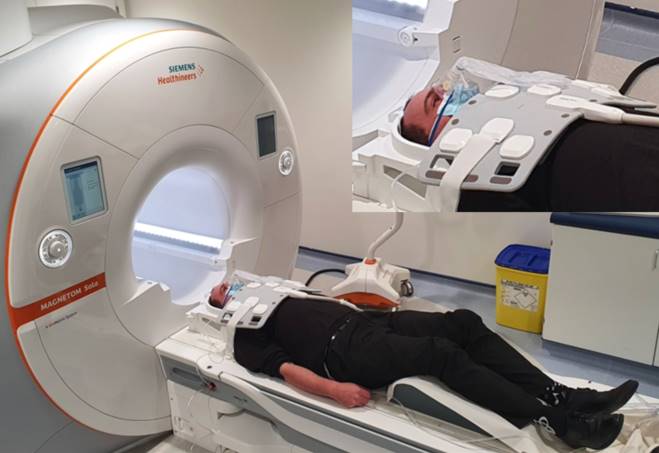


**Fig. S1.** A member of the research team modelling the participant setup. The posterior component of the head coil was used together with an ultraflex large 18-channel coil positioned over the neck region allowing ready placement of the non-rebreather oxygen mask. The non-rebreather bag was positioned on top of the ultraflex coil. Study participants wore ear plugs and ear defenders and did not wear a surgical face mask during the scan.


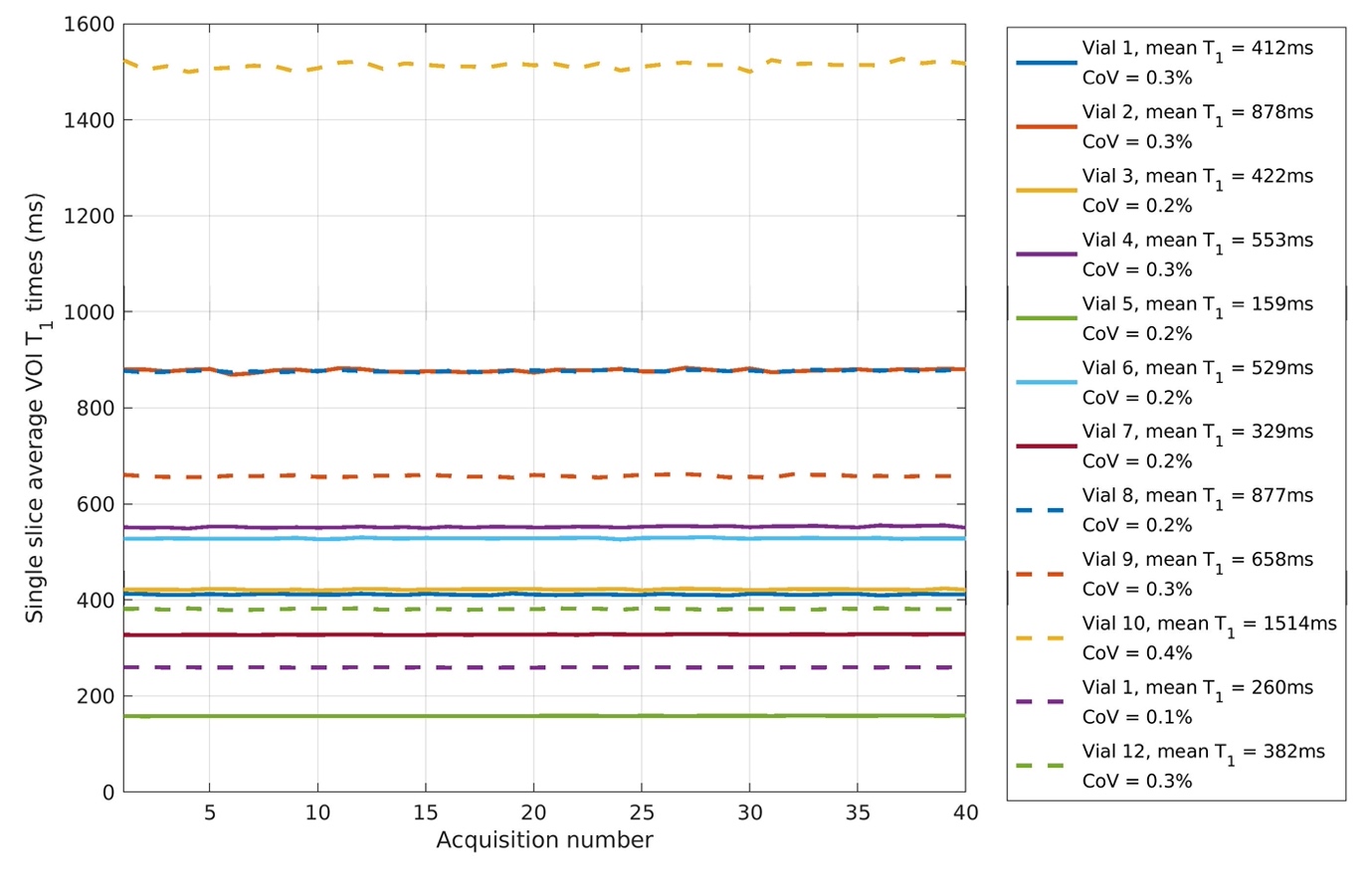


**Fig. S2.** T1 values for 12 tubes in a Eurospin TO5 phantom measured using the study dynamic vibe sequence. T1 values quoted are mean values over all 40 acquisitions. Coefficient of variations quoted for each tube have a median value of 0.2%.


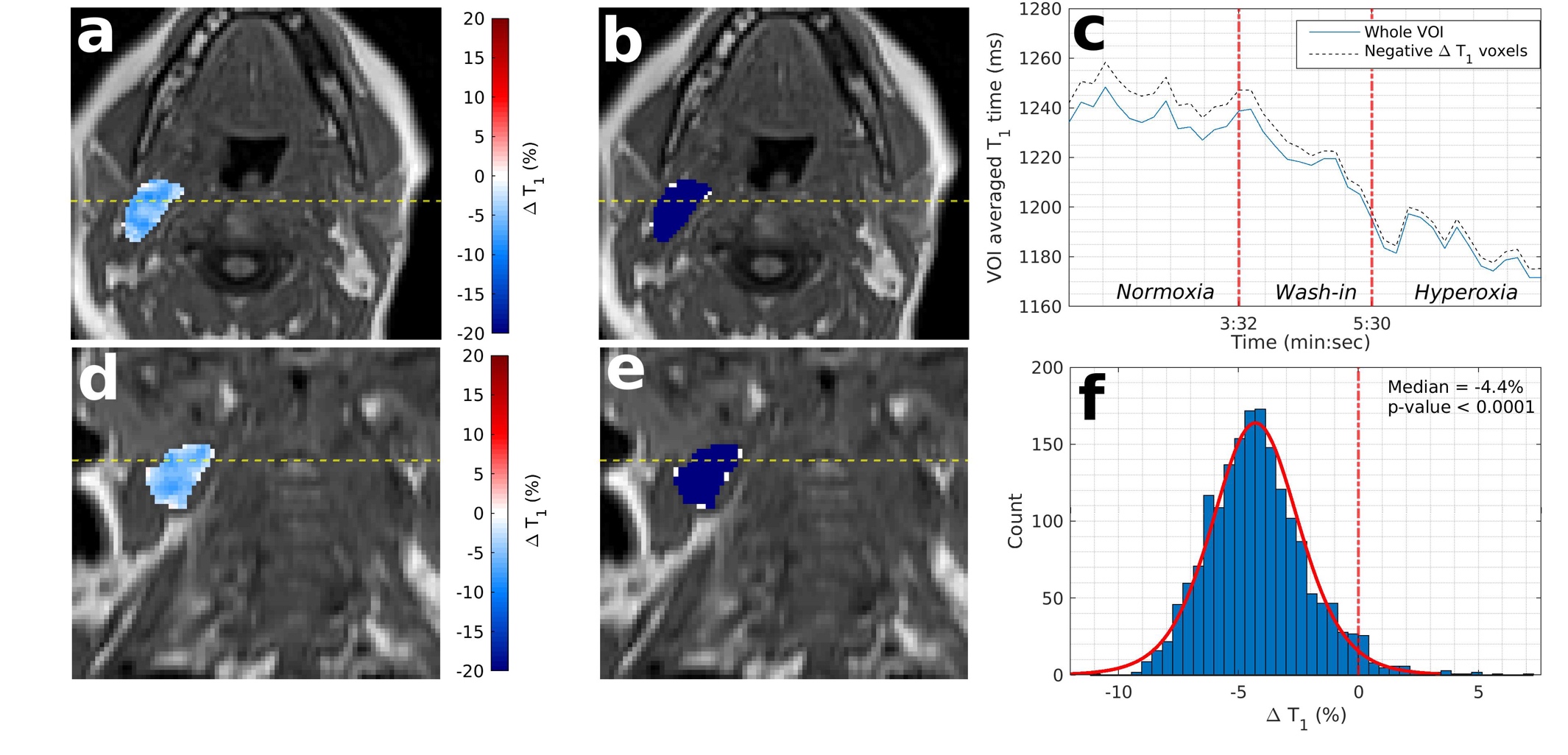


**Fig. S3.** Example OE-MRI parametrical maps in a patient with a suspected malignant nodal mass (patient no: 16 Lymph Node no: 2). Low estimated hypoxic fraction (11.6%) is shown on a T1 weighted vibe image with overlaid parametric ΔT1 map of the malignant mass (**a**, **d,** axial and coronal plane respectively) and overlaid statistical map of ΔT1 times (**b**, **e**). Blue colour indicates statistically significant decrease in T1 times, white indicating no statistically significant change and red indicating statistically significant increasing T1 times. **c** Time series of T1 times averaged over the entire malignant node VOI and over those voxels with significantly decreasing T1 times only. **f** Histogram of ΔT1 times for the entire malignant nodal VOI. *OE-MRI* Oxygen-enhanced magnetic resonance imaging, *VIBE* Volumetric interpolated breath-hold examination, *VOI* Volume of interest.

*
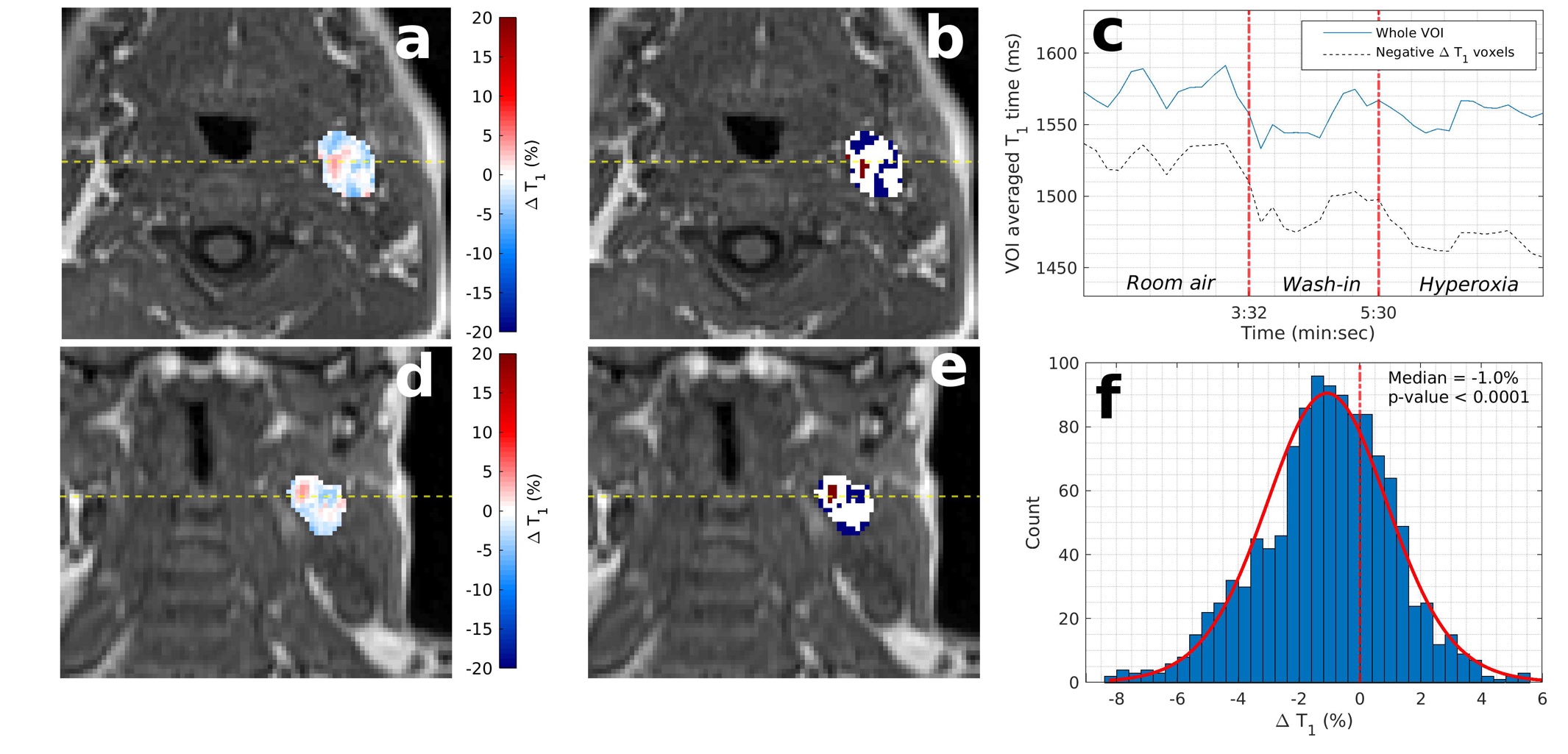
*

**Fig. S4.** Example OE-MRI parametrical maps in a patient with a suspected malignant nodal mass (patient no: 15 Lymph Node no: 7). High estimated hypoxic fraction (72.0%) is shown on a T1 weighted vibe image with overlaid parametric ΔT1 map of the malignant mass (**a**, **d,** axial and coronal plane respectively) and overlaid statistical map of ΔT1 times (**b**, **e**). Blue colour indicates statistically significant decrease in T1 times, white indicating no statistically significant change and red indicating statistically significant increasing T1 times. **c** Time series of T1 times averaged over the entire malignant node VOI and over those voxels with significantly decreasing T1 times only. **f** Histogram of ΔT1 times for the entire malignant nodal VOI. *OE-MRI* Oxygen-enhanced magnetic resonance imaging, *VIBE* Volumetric interpolated breath-hold examination, *VOI* Volume of interest.

**Supplementary Table**

**Supplementary Table *S*1** Summed scores from all patient participants for each of the 15 domains assessed in the MRI specific anxiety questionnaire.

|  | ***Total score for 20 patient participants*** | | |
| --- | --- | --- | --- |
| **Statement** | **Mask on** | **No mask** | ***Difference*** |
| 1. I felt that I controlled the situation | 27 (73) | 24 (76) | *-3* |
| 2. I had palpitations (heart feeling unusual) | 24 | 22 | *-2* |
| 3. I found it hard to breathe | 22 | 23 | *1* |
| 4. I was afraid | 32 | 27 | *-5* |
| 5. I wanted to come out | 26 | 27 | *1* |
| 6. I panicked | 22 | 22 | *0* |
| 7. I felt relaxed | 36 (64) | 29 (71) | *-7* |
| 8. I felt safe | 25 (75) | 24 (76) | *-1* |
| 9. I worried in advance | 35 | 28 | *-7* |
| 10. I felt calm | 31 (69) | 27 (73) | *-4* |
| 11. I had to force myself to manage the situation | 31 | 30 | *-1* |
| 12. Self-control was required when going through the examination | 38 | 35 | *-3* |
| 13. I needed support and encouragement | 25 | 25 | *0* |
| 14. I wished to have someone with me | 26 | 26 | *0* |
| 15. I needed more detailed information | 20 | 21 | *1* |

Lowest potential summed score is 20 (lowest anxiety) to 80 (maximum anxiety). The scores for domains 1, 7, 8 and 10 have been inverted to allow ease of comparison (original scores stated in brackets).
